# The Evaluation of Web-Based Communication Interventions to Support Decisions About COVID-19 Vaccination Among Patients With Underlying Medical Conditions: Protocol for a Randomized Controlled Trial

**DOI:** 10.2196/42837

**Published:** 2023-01-19

**Authors:** Minjung Lee, Bumjo Oh, Nan-He Yoon, Shinkyeong Kim, Young-Il Jung

**Affiliations:** 1 Institute of Health and Environment Seoul National University Seoul Republic of Korea; 2 Department of Dental Education Seoul National University Seoul Republic of Korea; 3 Department of Family Medcine SMG-SNU Boramae Medical Center Seoul Republic of Korea; 4 Division of Social Welfare and Health Administration Wonkwang University Ik-san Republic of Korea; 5 Department of Public Health Sciences Graduate School of Public Health Seoul National University Seoul Republic of Korea; 6 Department of Environmental Health Korea National Open University Seoul Republic of Korea

**Keywords:** COVID-19, vaccine, decision-making, randomized controlled trial, patient decision aid

## Abstract

**Background:**

The timeliness of raising vaccine acceptance and uptake among the public is essential to overcoming COVID-19; however, the decision-making process among patients with underlying medical conditions is complex, leading individuals to vaccine hesitancy because of their health status. Although vaccine implementation is more effective when deployed as soon as possible, vaccine hesitancy is a significant threat to the success of vaccination programs.

**Objective:**

This study aims to evaluate the effectiveness of a communication tool for patients with underlying medical conditions who should decide whether to receive a COVID-19 vaccine.

**Methods:**

This 3-arm prospective randomized controlled trial will test the effect of the developed communication intervention, which is fully automated, patient decision aid (*SMART-DA)*, and user-centered information (*SMART-DA*-α). The web-based intervention was developed to help decision-making regarding COVID-19 vaccination among patients with underlying medical conditions. Over 450 patients will be enrolled on the web from a closed panel access website and randomly assigned to 1 of 3 equal groups stratified by their underlying disease, sex, age, and willingness to receive a COVID-19 vaccine. *SMART-DA*-α provides additional information targeted at helping patients’ decision-making regarding COVID-19 vaccination. Implementation outcomes are COVID-19 vaccination intention, vaccine knowledge, decisional conflict, stress related to decision-making, and attitudes toward vaccination, and was self-assessed through questionnaires.

**Results:**

This study was funded in 2020 and approved by the Clinical Research Information Service, Republic of Korea. Data were collected from December 2021 to January 2022. This paper was initially submitted before data analysis. The results are expected to be published in the winter of 2023.

**Conclusions:**

We believe that the outcomes of this study will provide valuable new insights into the potential of decision aids for supporting informed decision-making regarding COVID-19 vaccination and discovering the barriers to making informed decisions regarding COVID-19 vaccination, especially among patients with underlying medical conditions. This study will provide knowledge about the common needs, fears, and perceptions concerning vaccines among patients, which can help tailor information for individuals and develop policies to support them.

**Trial Registration:**

Korea Clinical Information Service KCT0006945; https://cris.nih.go.kr/cris/search/detailSearch.do/20965

**International Registered Report Identifier (IRRID):**

DERR1-10.2196/42837

## Introduction

### Background

Given the large-scale impact, timing, and unpredictability of the COVID-19 pandemic and the threat this has posed to the health care system's routine capabilities, the battle against COVID-19 is still ongoing. The development of effective vaccines was highly anticipated, and several are now available. The timeliness of raising vaccine acceptance and uptake among the public is essential to overcoming COVID-19. A sizable proportion of the population must be vaccinated to achieve herd immunity and prevent the continued spread of the virus. When COVID-19 vaccination was first implemented, the government of the Republic of Korea specified that herd immunity would be achieved by November 2021 and proposed the goal of vaccinating 70% of the population by the end of October 2021 [1]. Recent studies have shown that third shots (boosters) of COVID-19 vaccines effectively provide additional protection from infection, and some countries are now offering fourth doses [2].

The decision to receive a vaccine is complex and reflects one’s values, attitudes, and experiences and the information one receives about vaccination. Although vaccine implementation is more effective when deployed as soon as possible, vaccine hesitancy is a substantial threat to the success of vaccination programs [3-5]. A well-known aspect of COVID-19 vaccination is that it may induce side effects. Data on the short-term and long-term side effects of vaccination have been reported [6,7], raising concerns regarding these side effects [8]. Although possible severe side effects are comparatively rare, the risk of their occurrence may considerably influence one’s decision not to receive the vaccine, thereby leading to vaccine hesitancy [9,10].

For patients with underlying diseases, the decision process becomes even more complex, ultimately leading them to avoid vaccination owing to the risk of harming their health [10,11]. In Korea, individuals with underlying diseases are classified as high risk and selected as the priority demographic for vaccination [1]. Previous studies have shown inconsistent COVID-19 vaccination intentions among populations with medical conditions [12,13]. Korea Centers for Disease Control and Prevention data from 2021 show that patients with chronic diseases had a lower vaccination rate than the general population of Korea. More recent data from May 2022 show that although the vaccination rate among those aged 40 to 59 years in the general population is 96.7%, that among patients with chronic disease is approximately 2% to 7% lower (94.7% among patients with hypertension, 93.7% among patients with diabetes, and 90.3% among patients with solid organ malignancy) [14]. A study in Brazil showed that concurrent malignancy, fibromyalgia, hydroxychloroquine use, and recent corticosteroid pulse therapy were independently associated with higher odds of COVID-19 vaccine hesitancy [13]. Conversely, a Japanese study revealed that a higher COVID-19 vaccine rate was positively associated with underlying disease, as patients with underlying diseases perceived themselves as being more vulnerable to COVID-19 [12].

Communication interventions such as decision aids (DAs) may yield promising results in addressing this issue. The International Patient Decision Aid Standards (IPDAS) Collaboration defines DAs as “tools designed to help people participate in decision making about health care options” [15]. They provide information on different health care options and help patients by clarifying and communicating personal values while guiding them through the decision-making process [16]. DAs aim to include high-quality epidemiological evidence on the benefits, risks, and associated probabilities of outcomes [17]. Their most common forms are web-based tools, pamphlets, and videos, and they fall within the broader field of shared decision-making—a process by which patients and clinicians work together to (1) clarify treatment goals and (2) review information about the available options and their outcomes to reach a mutual agreement [18,19]. DAs have improved patients’ perceptions of the decision-making process and increased their knowledge about the options that align with their values [20]. Although they effectively assist patients in making health treatment and screening decisions, less is known about the impact of DAs on vaccination uptake. A study that examined the impact of DAs on health care personnel’s decisions regarding immunization against influenza [21] and hepatitis B [22] found that DAs could be good tools for addressing the phenomenon of vaccine hesitancy [19,23].

### Goal of This Study

Several intervention studies attempting to increase the acceptance of COVID-19 vaccination via informational messages, behavioral nudges, web-based interventions, and videos appear in the academic literature [24-28]. However, interventions targeting patients with underlying medical conditions could not be uncovered in the literature. Therefore, the implementation of interventions with proven effectiveness targeting patients with underlying medical conditions are highly needed [29].

This study aims to evaluate the effectiveness of a communication tool for patients with underlying medical conditions for deciding on whether to receive a COVID-19 vaccine. This study develops a web-based communication intervention called “*SMART*,” a COVID-19 vaccination DA, and a vaccine-related information program tailored to the target the population with underlying diseases.

## Methods

### Study Design

**Figure 1:**
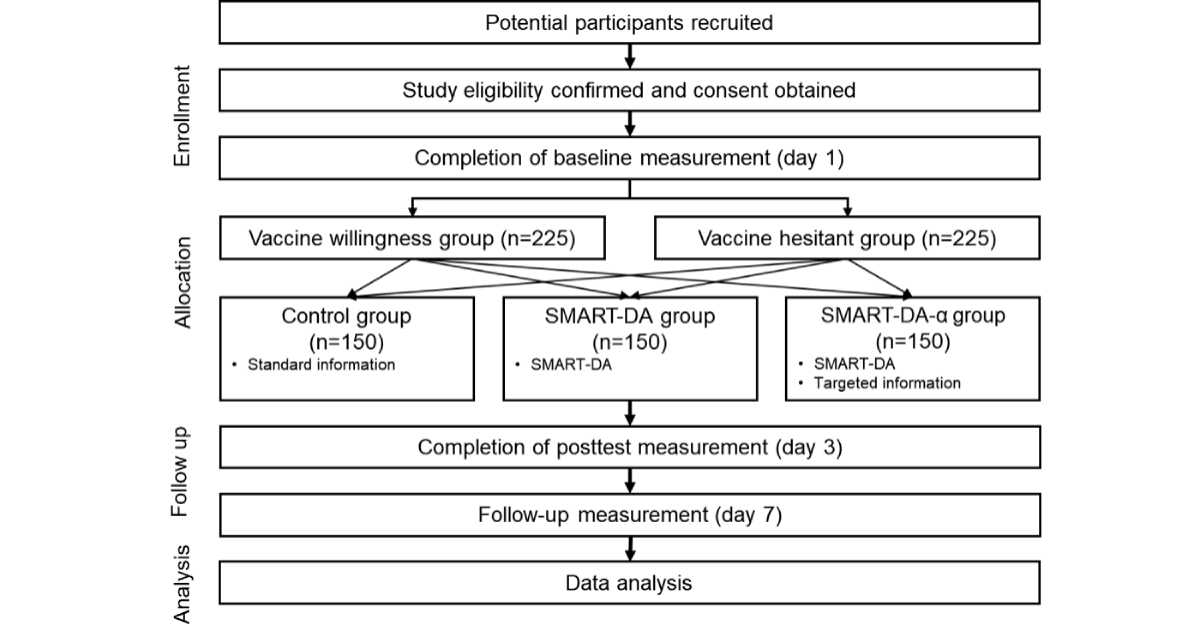
Overview of the stratified randomized controlled trial.

A randomized controlled trial (RCT) will be conducted and reported according to the CONSORT-EHEALTH (Consolidated Standards of Reporting Trials of Electronic and Mobile Health Applications and Online Telehealth) checklist, and the protocol timeline is shown in Table 1. This study is a 3-arm prospective RCT enrolling >450 participants with underlying medical conditions (Figure 1). The participants who complete their baseline web-based questionnaire will be recruited into the study and randomly assigned to 3 equal groups, stratified by their underlying disease, sex, age, and willingness to receive a COVID-19 vaccine (willingness and hesitation). The participants will be randomized to receive (1) standard information about COVID-19 vaccination (control group), (2) COVID-19 vaccination DA (intervention group *SMART-DA*), or (3) COVID-19 vaccination DA with targeted information provision (intervention group *SMART-DA*-α). Each group is described in detail in the following sections. After 1 week, we will follow-up with the participants and use an intention-to-treat (ITT) approach to determine the effects of their COVID-19 communication intervention on COVID-19 vaccination. There is no expected harms or unintended effects in each group.

**Table 1:** Spirit figure protocol timeline.

			Enrollment		Pretreatment assessment		Allocation		Postallocation assessment				Close out
Time point			-t_1_		0		0		t_1_		t_2_		t_3_
**Enrollment**													
	Eligibility screen	✓											
	Informed consent	✓											
	Allocation					✓							
**Interventions**													
	*SMART-DA*							✓					
	*SMART-DA-α*							✓					
**Assessments**													
	Sociodemographics			✓									
	Health-related factors			✓									
	Health literacy			✓									
	COVID-19 vaccination intention			✓						✓		✓	
	Knowledge about COVID-19 vaccination			✓						✓		✓	
	Decisional conflict			✓						✓		✓	
	Stress related to COVID-19 vaccination			✓						✓		✓	
	Perceived benefits of COVID-19 vaccination			✓						✓		✓	
	Perceived barriers to COVID-19 vaccination			✓						✓		✓	

### Participants and Setting

Recruitment will be conducted on the web to reflect the web-based nature of the intervention. The intervention study will be conducted via a web-based platform from a research company. The company will recruit participants by sending study invitations via emails or SMS text messages containing general information about the study (eg, the study aims and consent statement), and a URL to access the platform to survey panel members who meet the inclusion criteria. The inclusion criteria are as follows. First, the participants must have received a diagnosis of at least one of the following diseases: hypertension, diabetes, cancer, angina, myocardial infarction, or stroke. Second, the participants must be aged between 18 and 70 years. Third, the participants must have a fluent in the Korean language and be a resident of the Republic of Korea. Fourth, the participants must have access to the internet and the necessary internet literacy skills to use the interventions. Finally, those who have completed the COVID-19 vaccination (those who have received 2 or more doses and those who have been revaccinated regularly) will be excluded. Upon meeting all the inclusion criteria of the study, the participants will provide their informed consent electronically. The informed consent will appear on the study’s first page, and the research company will ensure the confidentiality of all the participants. The participants will be informed that they may discontinue participation in the research study at any time without any penalty or loss of benefits. All the researchers in this project will be given access to the cleaned data sets, and all data sets will be password protected. To ensure confidentiality, the data dispersed to the project team members will be blinded of any identifying participant information.

### Study Procedures and Implementation Strategies

#### Randomization

A representative sample of 450 individuals will be randomly selected through a proportional stratified sampling method based on sex and age from a list of candidates registered with the research company. The D-optimal blocking procedure proposed in a recent study [30] will be used for random assignment to reduce interference on the intervention effect by covariates such as the individual’s underlying disease, sex, age, and intention to receive COVID-19 vaccination. D-optimality is based on the determinant of the information matrix for the design, which is the same as the reciprocal of the determinant of the variance-covariance matrix for the least squares estimates of the linear parameters of the model. The D-optimal blocking procedure uses a SAS/QC procedure OPTEX (version 9.4; SAS Institute) to randomly allocate the participants to 1 of the 3 arms by all the researchers. The assigned participants will be blinded so that they do not know whether they belong to the intervention group or control group. Both the assigned participants and data analysts will be blinded, and the data analysts will conduct effect analysis of the anonymized groups 1, 2, and 3 without being aware of whether the group is an experimental or control group. However, because the researcher providing the intervention is aware of which group the participants belong to, the study is single blind.

#### Intervention Group A: COVID-19 Vaccination DA (SMART-DA)

##### Initial Development of the COVID-19 Vaccination DA (SMART-DA)

The development process of the *SMART-DA* was based on the IPDAS guidelines for DA development. In line with these guidelines, the needs of end users and clinicians were assessed. In addition, we asked public health education experts for additional input. For the end users’ needs assessment, 20 interviews were used to determine end users’ needs regarding COVID-19 vaccination decision-making that should be described in the DA. The input of these qualitative studies informed the design and content of the DA, supplemented by IPDAS background papers, other relevant literature in the field, and consultation with established experts.

Next, a prototype was developed and alpha tested with 30 potential end users and 5 public health experts. During the alpha test, the participants were asked to focus on the content of the DA. Before the beta test, the comprehensibility of the DA text for people with limited health literacy was evaluated by 3 coauthors. On the basis of this evaluation, some sections and sentences were rephrased or simplified.

##### Usability Testing of SMART-DA

After the alpha test, the DA was beta tested among 10 medical experts and 10 potential end users. The participants were given access to the DA to assess its usability using the heuristic evaluation method (medical experts) and think-aloud method (potential end users). Using the heuristic evaluation method, the medical experts were asked to evaluate the DA against a list of recognized usability principles: (1) uses simple and natural dialogue, (2) is in the user’s language, (3) minimizes memory load, (4) is consistent, (5) provides feedback, (6) offers clearly marked exits, (7) includes shortcuts, (8) provides helpful error messages, (9) prevents errors, and (10) provides help and documentation. Using the think-aloud method, the end users used the DA while verbalizing their thoughts. The think-aloud method is particularly useful in understanding the processes of cognition and is of high value in evaluating an intervention’s design for usability flaws [31]. The data gathered through these tests are compared and compiled into a summary describing the usability flaws of the DA.

##### Description of SMART-DA

The *SMART-DA* follows a stepwise approach and encourages decisions consistent with the participant’s values and health condition. The *SMART-DA* consists of five steps: (1) clarify the decision; (2) explore the decision in terms of knowledge, values, and certainty; (3) clarify decisional support; (4) identify the decision-making needs; and (5) plan the next steps based on the needs.

Step 1 consisted of 4 questions that required the participants to consider the key decision. The following questions were addressed: (1) “What options are available when deciding to be vaccinated against COVID-19?” (2) “What is making your COVID-19 vaccination decisions difficult?” (3) “When do you think it is time to make a COVID-19 vaccination decision?” and (4) “How far have you reached the final decision on the COVID-19 vaccination?” Multiple options were provided to increase the participants’ convenience. For question 1, we provided 3 options for COVID-19 vaccination decision-making: “Get vaccinated as soon as possible,” “Delay vaccination as much as possible,” and “Refuse vaccination.” If necessary, they could write their responses in the “other” column.

In step 2, the participants explored their knowledge and values related to each option to clarify how certain they were in their decision-making. A list of reasons to choose and not to choose each option (eg, “Get vaccinated as soon as possible,” “Delay vaccination as much as possible,” and “Refuse vaccination”) was provided, and the participants rated how much each reason mattered to them using a 6-point Likert-type scale. If necessary, they could write their reasons in the “other” column and rate how much they mattered. After the rating was completed, the total score for choosing each option and the reasons for not choosing it were calculated and shown. The participants were then asked what their preferred options were.

In step 3, the participants were asked to think about who supports their decision-making and how the support affects them. We asked them to identify whether other people supported their decision-making and, if so, who those individuals were. In addition, we asked them what role they preferred to play in decision-making and provided the response options “share the decision,” “decide myself after hearing views,” and “someone else decides.”

In step 4, the participants identified their decision-making needs concerning knowledge, values, support, and certainty. The participants considered whether (1) they had adequate knowledge of the risks and benefits of each option (knowledge), (2) they identified their values related to the risks and benefits of each option (value), (3) they had sufficient support to make choices (support), and (4) they were certain of their choices (certainty). The responses were collected with a yes or no option; if the participants had ≥1 “no” responses, they were encouraged to start over from step 2.

In the final step, the participants received information about the actions they could perform based on their decision-making needs. For instance, if the value of each option was unclear, we recommended revising the scores given in step 2 after listening to others’ experiences of COVID-19 vaccination or asking for advice from others.

As shown in Figure 1, the intervention group A is supplemented with *SMART-DA*. The participants were allowed to access and use *SMART-DA* whenever available, and as much as needed.

#### Intervention Group B: COVID-19 Vaccination DA With Targeted Information (SMART-DA-α)

As shown in Figure 1, the intervention group B is supplemented with targeted information in addition to the contents of intervention group A. The participants were allowed to access and use *SMART-DA*-α whenever available, and as much as needed. An educational program was planned to provide accurate and targeted information on COVID-19 vaccination to support decision-making among patients with underlying medical conditions. Educational material was developed with a tailored message to enhance the participants’ intuitive understanding and provide information to those who hesitated to make decisions on vaccination. Tailored messages were developed based on the participants’ interests in and perception of COVID-19 vaccination identified from the interview, such as their perception of COVID-19 vaccine safety, efficacy, and side effects. The findings revealed that the main messages in the educational material should be as follows:

1. COVID-19 vaccination: How effective is it?
2. COVID-19 vaccination: Is it safe?
3. Refusal to be vaccinated: critical cases

Tailored messages targeting the information needs of patients with underlying medical conditions were developed and structured around actual cases in clinical settings.

#### Control Group

The participants in the control group will receive standard information about COVID-19 vaccination provided by the Korean Disease Control and Prevention Agency. This easy-to-understand information about COVID-19 vaccination is publicly available. The material is accessible on the Korean Disease Control and Prevention Agency web page or YouTube (Google LLC) [32].

### Follow-up Measurements

After the intervention, the participants will complete follow-up surveys, 3 and 7 days after the intervention, amounting to a total of 3 surveys during the study period. The participants in all the study groups will receive automated text message notifications to complete their surveys to improve study retention.

### Incentives

The participants will be able to earn up to KRW 15,000 (approximately US $13) in gift cards for completing the study’s requirements. These incentives include a gift card worth KRW 5000 (US $4.01) for completing the initial baseline survey and engaging with the SMART-DA or SMART-DA-α intervention or control group material on the web; a gift card worth KRW 5000 for completing the 3-day survey; and a gift card worth KRW 5000 for completing the 7-day survey. All the incentives will be sent to the participants via their mobile phones.

### Measures

#### Participant’s Characteristics

Sociodemographic factors include sex (1=male and 2=female), age, family size (ie, living alone or with 1 or more person), the presence of children at home who attend school (more than one=1 and none=0), marital status (ie, married, single, divorced, or bereaved), and residence (urban=1 and rural=2). We will also assess educational level (1=middle school or below, 2=high school graduate, and 3=college and above) and monthly household income in KRW (1=<KRW 200 million, 2=KRW 200 million to 399 million, 3=KRW 400 million to 599 million, and 4=KRW ≥600 million).

Health-related factors include the respondent’s seasonal influenza vaccination history, underlying diseases, and prior COVID-19 diagnosis. For seasonal influenza vaccination history, the participants will be asked, “Have you been vaccinated against the seasonal influenza flu in the last five years?” The responses include “every year,” “more than once,” “maybe once,” “never,” and “don’t know.” We will also investigate the presence of underlying diseases by asking the participants to indicate all diagnosed underlying diseases (eg, hypertension, dyslipidemia, diabetes, chronic cardiac disease, asthma, and cancer). The participants who have not been diagnosed with underlying diseases are excluded from further study.

To examine health literacy related to COVID-19, we adapt the HLS-COVID-Q22 that has been translated into Korean. The HLS-COVID-Q22 was developed in Germany in 2020 by Okan et al [33] based on the European Health Literacy Survey Questionnaire. Its validity and reliability were verified among the German population aged ≥16 years (α=.94; *ρ*=0.891) [33]. The HLS-COVID-Q22 contains 22 items organized into 4 subgroups: accessing (6 items), understanding (6 items), appraising (5 items), and applying (5 items) health-related information in terms of COVID-19. It is answered on a 4-point scale, ranging from “4=very easy” to “1=very difficult.”

#### Primary and Secondary Outcomes

The primary and secondary outcomes and potential mediators are listed in Table 2. Our primary outcome is COVID-19 vaccination intention, which will be an important outcome during the 1-week follow-up period. The baseline, posttest (3-day), and follow-up (1-week) surveys will assess the participants’ vaccination intention. A 5-point Likert scale questionnaire will measure the intention to be vaccinated against COVID-19. The participants will be asked, “If a vaccine for Coronavirus (COVID-19) becomes available, would you want to receive it?” and provided the response options “1=Definitely Would Not Want It, 2=Would Likely Not Want It, 3=Neutral or Unsure, 4=Would Likely Want It, and 5=Definitely Would Want It.” The level of vaccine acceptance is defined as willing (“Definitely Would Not Want It,” and “Definitely Would Want It”) or hesitant (“Definitely Would Not Want It,” “Would Likely Not Want It,” and “Neutral or Unsure”). The secondary outcomes include knowledge level related to COVID-19, which will be assessed using a validated questionnaire that has been used in numerous studies [34,35], and decisional conflict, which will be assessed using the decisional conflict scale [36]. In addition, psychological stress owing to decision-making regarding COVID-19 vaccination will be measured.

**Table 2:** Study outcome measures.

			Baseline measurement (day 1)		Posttest measurement (day 3)		Follow-up measurement (day 7)
**Primary outcome**							
	COVID-19 vaccination intention	✓		✓		✓	
**Secondary outcomes**							
	Knowledge about COVID-19 vaccination	✓		✓		✓	
	Decisional conflict	✓		✓		✓	
	Stress related to COVID-19 vaccination	✓		✓		✓	
	Perceived benefits of COVID-19 vaccination	✓		✓		✓	
	Perceived barriers to COVID-19 vaccination	✓		✓		✓	

### Data Collection and Management

We collected quantitative data on the potential mediators of implementation effects, including attitudes toward COVID-19 vaccination (eg, perceived benefits and barriers). Questionnaires which were validated for web-based use and apply Checklist for Reporting Results of Internet E-Surveys items were designed to measure primary and secondary outcomes. Data collection is performed according to standard guidelines. To improve the reliability of data quality, real-time support through instant messages or phone calls regarding data collection were provided during the entire data collection period. The respondents entered a web-based data collection program, self-assessed through web-based questionnaires, which the research team reviews and rechecks for anomalous responses according to a defined monitoring strategy. If an error was found in the data, the research team asked the rater to correct or explain it, and all corrections were made after the research team confirms them. In addition, to facilitate retention, respondents reported any issues with the respondent. In the event of study discontinuation, a report was generated in a concise format. All available data were checked by the research team members. Every respondent was assigned a unique study ID, and the research team trained the researchers to protect the study data to ensure data safety. The data collection form does not contain any personally identifiable information. The password-protected electronic files were stored on a password-protected computer. Data managers and analysts independently conducted inspections for each evaluation.

### Sample Size and Power

We sought to detect the main effects of the communication intervention within the 3 groups with at least 90% power using a 2-sided α of .05. We used G*Power (latest version 3.1.9.7; Heinrich-Heine-Universität Düsseldorf) to calculate the sample size for 3 repeated measurements (in-between interaction, small effect size of 0.2). Our target analytic sample size was 450 (150 per study group) participants. To achieve this analytic sample size, we randomized a projected 630 participants (210 per study group) and expected approximately 30% of the initial participants to drop out during the 7-day follow-up period.

### Statistical Methods

#### Evaluation of Efficacy

We will also examine the descriptive statistics for the participants’ baseline characteristics and compare them across the 3 study groups. The main analysis will be performed based on the ITT principle, involving all the randomized participants to investigate the effectiveness of the interventions. The ITT principle can minimize potential bias owing to dropouts and can be applied while performing further preprotocol analyses. The effects of the interventions on the primary outcome (eg, vaccination intention) and secondary outcomes (ie, knowledge related to COVID-19 vaccines, decisional conflict, psychological stress related to vaccination, perceived benefits of COVID-19 vaccines, and perceived barriers to COVID-19 vaccines) will be estimated using linear mixed-effects models for continuous outcome variables and generalized linear mixed-effects models for categorical outcome variables. The test statistic will be calculated to assess any significant intervention benefit; we will use 2-sided *P* values with α=.05 to calculate the level of significance.

#### Subanalyses

We will perform a path analysis to examine the theoretical constructs as potential mediators following the effect evaluation. For each outcome, a path analysis will be performed to describe the direct and indirect relationships, including the perceived benefits of and barriers to the COVID-19 vaccine. Path models are statistical methods that, compared with multiple regressions, allow for the simultaneous assessment of several regression paths occurring between multiple dependent and independent variables and for the computation of direct, indirect (mediated), and total effects. Standardized parameter estimates (standardized β) will be used to compare the magnitude of associations between media use and mediators. The model parameters and SE estimates are to be calculated. The participants with missing data will be excluded from analysis. Statistical analyses will be conducted using STATA (version 15; StataCorp LLC), SAS (version 9.4; SAS Institute), and R (version 4.2; R Foundation for Statistical Computing) software.

In addition, we will conduct subgroup analyses according to the preintervention intent for vaccination. We will observe how the degree of vaccination intention changes in the 2 groups. Among those who have already been willing to receive a COVID-19 vaccine, there are cases in which their decisions are passive or involuntary owing to social pressure or economic activity. Therefore, we seek to identify whether the nature of their decisions changes through the intervention.

The Data Monitoring Committee comprises the Principal Investigator, independent external data managers, and statisticians. The committee is responsible for data quality throughout the data collection, analysis, and reporting processes. The Data Monitoring Committee is independent of the research funding agency and has no competing interests.

### Ethics Approval

Ethics approval for this study was obtained from the Korea National Open University’s institutional review board on December 1, 2021 (IRB number ABN01-202111-21-13), and was registered with the Clinical Research Information Service Registry. The current protocol version is version 1.1, and there is no plan to change it. The risk of negative effects on participant outcomes is minimal. The possibility of SMART-DA not having a positive effect on the participants is present, but the risk of negative participant outcomes because of this is limited. The data will be analyzed at the completion of the trial, and the study findings will be published in major peer-reviewed journals. Upon meeting all the inclusion criteria of the study, all the study participants will provide their informed consent electronically.

## Results

Data were collected from December 2021 to January 2022. This paper was initially submitted before data analysis began. The results are expected to be published in the winter of 2023.

The number of individuals who initially expressed their intention to participate was 1066. Of these 1066 individuals, 515 (48.31%) people participated in the first survey, excluding 551 (51.69%) people who did not meet the inclusion criteria. All dropouts were individuals without underlying diseases. Of the 515 participants in the first survey, 466 (90.5%) participated in all 3 rounds and 69 (13.4%) dropped out. No significant differences were observed in sex, age group, residence area, education level, marital status, or willingness to receive a COVID-19 vaccine between those who participated in all 3 surveys and those who dropped out based on the first survey (Table 3). The results of the chi-square test conducted to confirm the distribution bias of the participants assigned to the intervention groups (SMART-DA and SMART-DA-a) and those assigned to the control group, in terms of sex, age group, region of residence, education, marital status, and intention to receive a COVID-19 vaccine, showed that no significant differences were observed (Table 4). Among the 515 individuals who completed the first survey, 294 (57.1%) were male and 221 (42.9%) were female, and the age distribution was even (18-29 years, n=90, 17.5%; 30-39 years, n=98, 19%; 40-49 years, n=124, 24.1%; 50-59 years, n=123, 23.9%; 60-69 years, n=80, 15.5%). The rate of college education or higher (418/515, 81.2%) was higher than that of high school graduation (97/515, 18.8%), and the most common marital status was having a spouse (310/515, 60.2%). The proportion of those who responded that they had low or no intention of receiving a COVID-19 vaccine was 46% (237/515), and the proportion of those who responded that they had high intention of receiving a COVID-19 vaccine was 53.9% (278/515), indicated a rather high initial willingness among the participants to receive a COVID-19 vaccine.

**Table 3:** Comparison of the participants who responded 3 times with those who responded <3 times (N=515).

			Incomplete response (<3 times; n=69), n (%)		Full response (3 times; n=446), n (%)		*P* value	
**Sex**								.10
	Male	33 (47.8)		261 (58.5)				
	Female	36 (52.2)		185 (41.5)				
**Age (years)**								.37
	18-29	10 (14.5)		80 (17.9)				
	30-39	12 (17.4)		86 (19.3)				
	40-49	16 (23.2)		108 (24.2)				
	50-59	23 (33.3)		100 (22.42)				
	≥60	8 (11.6)		72 (16.1)				
**Residence**								.53
	Urban	66 (95.7)		418 (93.7)				
	Rural	3 (4.3)		28 (6.3)				
**Education**								.81
	High school graduation or lower	11 (15.9)		86 (19.3)				
	College and above	58 (84.1)		360 (80.7)				
**Marital status**								.92
	Other (unmarried, widowed, or divorced)	26 (37.7)		179 (40.1)				
	With a spouse	43 (62.3)		267 (59.9)				
**Willingness to receive a COVID-19 vaccine**								.17
	Low	37 (53.6)		200 (44.8)				
	High	32 (46.4)		246 (55.2)				

**Table 4:** Basic characteristics of the participants at baseline (N=515).

			Total, n (%)		SMART-DA group (n=172), n (%)		SMART-DA-α group (n=172), n (%)		Control group (n=171), n (%)		*P* value	
**Sex**												.99
	Male	294 (57.1)		99 (57.6)		98 (57)		97 (56.7)				
	Female	221 (42.9)		73 (42.4)		74 (43)		74 (43.3)				
**Age (years)**												.93
	18-29	90 (17.5)		26 (15.1)		32 (18.6)		32 (18.7)				
	30-39	98 (19)		38 (22.1)		29 (16.9)		31 (18.1)				
	40-49	124 (24.1)		42 (24.4)		41 (23.8)		41 (24)				
	50-59	123 (23.9)		40 (23.3)		45 (26.2)		38 (22.2)				
	≥60	80 (15.5)		26 (15.1)		25 (14.5)		29 (17)				
**Residence**												.29
	Urban	484 (94)		162 (94.2)		158 (91.9)		164 (95.9)				
	Rural	31 (6)		10 (5.8)		14 (8.1)		7 (4.1)				
**Education**												.73
	High school graduation or lower	97 (18.8)		34 (19.8)		35 (20.3)		28 (16.4)				
	College and above	418 (81.2)		138 (80.2)		137 (79.7)		143 (83.6)				
**Marital status**												.57
	Other (unmarried, widowed, or divorced)	205 (39.8)		70 (40.7)		67 (39)		68 (39.8)				
	With a spouse	310 (60.2)		102 (59.3)		105 (61)		103 (60.2)				
**Willingness to receive a COVID-19 vaccine**												.99
	Low	237 (46)		79 (45.9)		80 (46.5)		78 (45.6)				
	High	278 (54)		93 (54.1)		92 (53.5)		93 (54.4)				

## Discussion

### Principal Findings

This study is conducted to evaluate the effectiveness of a web-based communication intervention in assisting people with underlying diseases in making decisions about whether to receive a COVID-19 vaccine. This RCT will test the effectiveness of the developed communication intervention, patient DA, and user-centered information as implementation strategies to help patients with underlying medical conditions in making decisions regarding COVID-19 vaccination. This communication intervention aimed to shed light on how a DA for patients with underlying diseases with decision-making needs about COVID-19 vaccination should be designed to facilitate an informed decision-making process. Moreover, this intervention study aimed to contribute to a deeper understanding of individuals’ preferences, attitudes, and values regarding COVID-19 vaccination and their impact on decisions.

A set of implications for interventions, communication strategies, and future research can be drawn from this study. First, the outcomes from this study provide valuable new insights into potential DAs for supporting informed decision-making regarding COVID-19 vaccination and discovering barriers to making informed decisions regarding COVID-19 vaccination, especially among patients with underlying medical conditions. Findings about what kind of information is especially misleading can help clinicians focus on these aspects in their consultations [37,38]. Moreover, knowledge about common needs, fears, and perceptions can help tailor information for individuals and develop policies to support them.

Second, this study is a critical step toward achieving the aim of increasing COVID-19 vaccination among patients with underlying diseases. It will comprehensively evaluate *SMART-DA* and *SMART-DA*-α. The results will explain the efficacy of the intervention and its potential mediators. If proven efficacious, *SMART-DA* could fill an important gap as a web-based intervention that allows patients to learn about COVID-19 vaccination and promotes vaccination by stimulating informed decision-making. Moreover, this intervention could be used not only to defeat the battle against COVID-19 but also to prepare for future epidemics. This tool could be potentially distributed on the web before and during the implementation of vaccination programs. As implementation planning is an important part of the process of implementing evidence-based practices [39], this study will enable public health authorities to use these tools as means of support for patients.

### Limitations

This study has several limitations. First, we could not investigate the study participants’ COVID-19 vaccination status as a primary outcome, as their medical records are not accessible. Therefore, we will investigate the participants’ self-reported willingness to receive a COVID-19 vaccine. However, there is a risk of bias in self-reported willingness and actual vaccination behavior. Second, our study participants might have different underlying medical conditions; therefore, the findings will not be generalizable to all patients. Third, the sample of this study is limited to certain underlying conditions; for example, it did not include patients who receive immunosuppressive therapy, who have a high need for COVID-19 vaccination. However, the diseases targeted in this study are major chronic diseases with high prevalence in Korea, for which the Korean government or medical society recommends vaccination against COVID-19. In addition, because the vaccination rate among this population is generally lower than that among the general population, it can be considered a meaningful group to study. Finally, because the recruitment of participants and implementation of the intervention are conducted through the internet, it is limited to those who have no problems using the internet. Therefore, the participation of those with limited internet use, especially older adults, is restricted; therefore, caution is required in interpretation. Finally, as participants are recruited through the internet, we limit participation to individuals aged <70 years, as those aged >70 years tend to have poorer control over internet use in Korea. Nonetheless, we acknowledge that the older adult group is an important group with high rates of underlying conditions and decision-making needs for which the development of alternative in-person modes of DA may be required in future studies.

### Conclusions

We initiated an intervention study to improve the situation in which people with underlying conditions requiring COVID-19 vaccination have lower vaccination rates compared with the general population. In this paper, we have outlined the study procedure and data management. This interventional study aimed to evaluate improvements in vaccination intention to control COVID-19. We plan to comprehensively analyze the data and continuously present new research to improve vaccination intentions.

## Abbreviations

CONSORT-EHEALTH: Consolidated Standards of Reporting Trials of Electronic and Mobile Health Applications and Online Telehealth
DA: decision aid
IPDAS: International Patient Decision Aid Standards
ITT: intention-to-treat
RCT: randomized controlled trial

## Data Availability

Data sharing does not apply to this study, as no data sets have been analyzed during this study. Data construction and analysis have been completed, and the data are available to the authors upon reasonable request and permission from the Korea National Open University’s institutional review board.
